# Delayed gastric emptying after Pancreaticoduodenectomy: a propensity score-matched analysis and clinical Nomogram study

**DOI:** 10.1186/s12893-020-00809-5

**Published:** 2020-07-09

**Authors:** Xianlei Cai, Miaozun Zhang, Chao Liang, Yuan Xu, Weiming Yu

**Affiliations:** Department of Gastrointestinal Surgery, Ningbo Medical Center Lihuili Hospital, 57 Xingning Road, 315000 Ningbo, PR China

**Keywords:** Pancreaticoduodenectomy, Delayed gastric emptying, Propensity score-matching, Nomogram

## Abstract

**Background:**

Delayed gastric emptying (DGE) is a common and frustrating complication of pancreaticoduodenectomy (PD). Studies suggest that surgical methods and other clinical characteristics may affect the occurrence of DGE. Nevertheless, the results of such studies are conflicting. The objective of this work was to perform a propensity score matching analysis to compare the differences between pylorus-preserving pancreaticoduodenectomy (PPPD) and pylorus-removing pancreaticoduodenectomy (PrPD) and to develop and validate a nomogram to predict the probability of severe DGE (SDGE).

**Methods:**

This retrospective study enrolled patients who underwent PD at our institution from December 2009 to December 2018. Propensity score matching was applied at a ratio of 1:1 to compare PPPD and PrPD groups. We compared incidence of complications, DGE, lengths of hospital stay, hospitalization costs, and mortality. Univariate and multivariate logistic regression analysis were performed to identify potential risk factors of severe DGE. Finally, a nomogram was developed and validated to predict severe DGE.

**Results:**

The PPPD group had a significantly higher rate of postoperative pancreatic fistula (29.9% versus 17.4%, *P* < 0.05) and less blood loss (463.7 ml versus 694.9 ml, *P* < 0.05). After propensity score matching, the PPPD group had a significantly higher rate of postoperative DGE (19.2% versus 3.8%, *P* < 0.05), especially severe DGE (17.3% versus 0%) than the PrPD group. There were no significant differences in terms of lengths of hospital stay, hospitalization costs or mortality between the groups. Surgical method, biliary leakage, abdominal infection, and diabetes were independent risk factors for SDGE. The nomogram predicted SDGE with a training C - index of 0.798 and a validation C - index of 0.721.

**Conclusion:**

PPPD increases the risk of DGE than PrPD, especially SDGE. Our prediction nomogram gives good prediction of SDGE after pancreaticoduodenectomy.

## Background

Pancreaticoduodenectomy (PD) is a common choice for treatment of benign lesions as well as malignant neoplasms in the periampullary region. The procedure includes a 30–40% distal gastrectomy [1] known as the conventional pancreaticoduodenectomy (cPD). Modifications of cPD have been reported, including subtotal stomach-preserving pancreaticoduodenectomy and pylorus- resecting pancreaticoduodenectomy [2, 3]. These are referred to as pylorus-removing pancreaticoduodenectomy (PrPD). Pylorus-preserving pancreaticoduodenectomy (PPPD) was first described by Traverso and Longmire [4] in 1978 with the preservation of the entire stomach; the procedure reduced the incidence of bile reflux gastritis and improved nutritional status [5, 6]. Nevertheless, delayed gastric emptying (DGE) became a common and frustrating complication after PPPD [7, 8]. Although DGE is not life threatening, it can prolong hospital stay and decrease the quality of life. Currently, debate continues as to which surgical procedure is superior.

Enhanced recovery after surgery (ERAS) pathways are used worldwide to optimize patient outcomes, shorten length of hospital stay and to reduce hospitalization costs [9, 10]. DGE is a huge obstacle for ERAS without compromising the safety of patients undergoing PD. Some meta-analyses have been performed [11, 12]; however, regarding DGE, high heterogeneity was found among pooled studies, and the standard of DGE and study design were not uniform; Furthermore, there are no tools to predict DGE after pancreatoduodenectomy; A nomogram would be a good tool for this purpose.

To compare possibility of postoperative DGE between PrPD and PPPD, and to develop a nomogram to predict incidence of severe DGE, we conducted this study based on a single center cohort.

## Methods

### Patient selection

This retrospective comparative study was performed at Ningbo Medical Center Lihuili Hospital in Zhejiang Province, China. Patients with pancreatic or periampullary lesions were identified from electronic medical records between December 2009 and December 2018. Exclusion criteria were as follows (1) tumor invading stomach tissue or had suspected metastasis of peripyloric lymph nodes or distant metastasis; (2) severe comorbidities prolonging the length of hospital stay, including heart failure, respiratory disorders, liver cirrhosis and mental illnesses. Comorbidities of the cardiovascular and cerebrovascular systems, respiratory system or nervous system can affect recovery, interfere with the short-term effect of the procedure and introduce bias. Finally, a total of 308 consecutive patients who underwent PD were identified (67 patients underwent PPPD and 241 patients underwent PrPD). We recorded medical histories, lab values, perioperative characteristics, postoperative outcomes, complications and mortality.

### Surgical procedures

Radical pancreatectomy and lymphadenectomy were performed for all patients by experienced surgeons. All the procedures were performed by Professor Caide Lu and his team. For PPPD, we divided the proximal duodenum 2 to 3 cm distal to the pylorus. For PrPD, the stomach was divided at 2 to 5 cm proximal to the pylorus. The surgical procedures were base the discretion of the surgeon rather than the extent of tumor or other factors. The peripyloric lymph nodes were also dissected in PPPD. Reconstruction was performed as follows: An end-to-side antecolic duodenojejunostomy in PPPD or gastrojejunostomy in PrPD was performed. An end-to-side pancreatojejunostomy or binding pancreaticogastrostomy [13] was chosen. An end-to-side choledochojejunostomy was performed both in PPPD and PrPD. The gastric outlet diameter of the anastomotic stoma was measured and confirmed intra-operatively.

### Postoperative management

All patients received proton pump inhibitor (PPI), prophylactic antibiotics, octreotide and nutritional support post-operatively. The nasogastric tube was removed within 3 days after the surgery according to the amount of drainage from the nasogastric tube. Oral fluid intake was started at the postoperative day 4–5 unless there were complications such as DGE. Other treatment would be given as indicated including prokinetic agents for abdominal discomfort and distention.

The consensus definition and clinical grading of DGE were proposed by the International Study Group of Pancreatic Surgery (ISGPS) [14]. DGE was classified into three categories (grades A, B and C) according to post-operative management, including the period of nasogastric tube required, reinsertion of nasogastric tube, the time of inability to tolerate oral intake of solids, vomiting / gastric distention or use of prokinetic agents [14]. Grades B and C usually required an adjustment of clinical management and was defined as severe DGE (SDGE). Other post-operative complications such as pancreatic fistula, intra-abdominal hemorrhage, and bile leakage were consistent with the definitions by the International Study Group [15–17].

### Propensity score matching

We conducted propensity score matching to control for confounding biases to construct a randomized experiment-like situation [18, 19]. Propensity scores (PS) were estimated using a logistic regression model [20] in which patient characteristics were regarded as covariates. These included age, gender, presence of diabetes mellitus, pre-operative albumin level, pre-operative leukocyte counts, total bilirubin level, jaundice reduction therapy (i.e. pre-operative stenting, percutaneous transhepatic cholangial drainage (PTCD), and endoscopic nasobiliary drainage (ENBD)), anastomotic pattern of the pancreas (pancreaticogastrostomy or pancreatojejunostomy), operative time, blood loss, enteral nutrition, and complications. Surgical methods were regarded as the dependent variable. We determined the degree of overlap PS value and covariates between PPPD and PrPD groups using propensity score graph. We conducted 1:1 matching using the nearest-neighbor method with a caliper of 0.02 to prevent bias from distant matches [21]. Balance between groups was defined as a *p*-value greater than 0.05.

### Statistical analysis

We compared the continuous variables using the Student t-test between PPPD and PrPD groups. The chi-square test or Fisher’s exact tests were performed for categorical variables. Univariate and multivariate logistic regression analyses were performed to calculate the odds ratios (ORs) and their 95% confidence intervals (CIs) for each risk factor for PPPD group with reference to the PrPD group. Inclusion and exclusion criteria of type I error = 0.10 were set in the stepwise multivariate logistic regression analysis.

Discrimination that reflected the ability of a predictive model to distinguish events and non-events correctly was validated using the concordance index (c-index), that is, a generalization of the area under the receiver operating characteristic (ROC) curve. Model calibration was validated using calibration plots and the Hosmer–Lemeshow method [22].

Propensity score matching was performed using STATA version 12.0 (StataCorp LP, College Station, TX, USA). Other statistical analyses were performed using IBM SPSS Statistics Software version 20.0 (IBM Corporation, Armonk, NY, USA) and R software for Windows, version 3.6.1. A *p*-value of < 0.05 (two-sided) was considered statistically significant.

## Results

### Patient characteristics

A total of 308 patients who underwent PPPD or PrPD in our hospital were identified from the electronic medical records, of which 199 (64.6%) were male and 109 (35.4%) were female. The mean age was 62.2 ± 11.5 years. The mean length of hospital stays was 20.3 ± 12.8 days. The incidence of DGE was 17.5% (54/308). Among all patients, 67 (21.8%) underwent PPPD and 241 (78.2%) underwent PrPD. We selected 52 pairs after 1:1 propensity score matching. Table 1 displays patient characteristics in the unmatched and the propensity score matched groups. The demographics were similar in the two groups. Table 2 displays the peri-operative data from both groups. In the unmatched group, patients in the PPPD group were more likely to undergo binding pancreaticogastrostomy and had less intraoperative blood loss when compared with the PrPD group. After propensity score matching, patient distributions between PPPD group and PrPD group were balanced.

**Table 1:** Characteristics of patients in unmatched group and the propensity score matched group

Parameter	Before propensity matching			After propensity matching		
	PPPD group (*n* = 67)	PrPD group (*n* = 241)	*P* value	PPPD group (*n* = 52)	PrPD group (*n =* 52)	*P* value
Age (years)	62.4 ± 13.0	62.1 ± 11.0	0.868	63.1 ± 13.2	63.4 ± 11.5	0.893
Gender (male/female)	37/30	162/79	0.069	30/22	24/28	0.326
Diabetes mellitus(%)	6(9)	35(14.5)	0.235	4(7.7)	3(5.8)	0.696
Albumin(g/dL)	38.7 ± 5.0	38.6 ± 4.8	0.891	38.2 ± 5.30	39.2 ± 5.07	0.352
WBC(/L)	6.4 ± 2.1	6.2 ± 2.3	0.595	6.25 ± 2.14	6.36 ± 2.03	0.797
TB (umol/L)	88.9 ± 96.0	98.9 ± 113.0	0.514	96.1 ± 98.9	96.5 ± 104.1	0.982
Malignant tumor(%)	62(92.5)	227(94.2)	0.575	47(90.4)	45(86.5)	0.760
Jaundice reduced(%)	5(7.5)	12(5)	0.544	5(9.6)	7(13.5)	0.539

*WBC* white blood cell, *TB* total bilirubin

**Table 2:** Operative factors of patients in unmatched group and the propensity score matched group

Parameter	Before propensity matching			After propensity matching		
	PPPD group (*n =* 67)	PrPD group (*n =* 241)	*P* value	PPPD group (*n =* 52)	PrPD group (*n =* 52)	*P* value
pancreaticogastrostomy (%)	55(82.1)	122(50.6)	0.000	42(80.8%)	41(78.8%)	0.807
Enteral nutrition(%)	9(13.4)	41(17.0)	0.482	9(17.3)	9(17.3)	1.000
Operation time (min)	358.6 ± 83.0	377.6 ± 90.0	0.125	353.5 ± 87.3	344.0 ± 56.8	0.512
Blood loss (ml)	463.7 ± 236.3	694.9 ± 680.9	0.012	471.7 ± 245.0	497.5 ± 279.7	0.618

Comparison of Short-Term Effects Between PPPD and PrPD.

Table 3 displays the incidence of complications, length of hospital stays, hospitalization costs, the proportion of death, and the incidence of DGE and severe DGE (grade B/C) in both groups. In the unmatched groups, the patients who underwent PPPD had a higher incidence of postoperative pancreatic fistula (29.9% versus 17.4%, *p* = 0.025), a shorter length of hospital stays (16.4 days versus 21.3 days, *p* = 0.004) and lower costs (41,273.2 CNY versus 48,869.8 CNY, *p* = 0.041) than did those who underwent PrPD, but they had higher incidence of severe DGE (17.9% versus 8.7%, *p* = 0.031). After propensity score matching, there was no significant difference in length of hospital stay or hospitalization costs between the PPPD and PrPD groups. However, patients in the PPPD group had a significantly higher risk of DGE and severe DGE than did patients in the PrPD group (*p* = 0.014 and *p* = 0.003, respectively). Before propensity matching, we found the selection of anastomotic pattern of the pancreas was significantly different between the PPPD and PrPD groups. In order to reduce the interference of this situation, we also analyzed the association between the anastomotic pattern of the pancreas and other clinical characteristics. The results indicated that the anastomotic pattern of the pancreas was not associated with blood loss and postoperative pancreatic fistula (*p* = 0.918 and *p* = 0.450, respectively; as shown in Supplementary Table 1).

**Table 3:** postoperative factors in unmatched group and the propensity score matched group

Parameter	Before propensity matching			After propensity matching		
	PPPD group (*n =* 67)	PrPD group (*n =* 241)	*P* value	PPPD group (*n =* 52)	PrPD group (*n =* 52)	*P* value
Complications(%)						
Pancreatic fistula	20(29.9%)	42(17.4%)	0.025	13(25.0%)	12(23.1%)	0.819
Biliary leakage	1(1.5%)	19(7.9%)	0.088	0(0%)	1(1.9%)	0.315
Intra-abdominal infection	19(28.4)	58(24.1%)	0.473	16(30.8%)	9(17.3%)	0.108
Bleeding	3(4.5%)	7(2.9%)	0.458	3(5.8%)	1(1.9%)	0.308
hospital stay (day)	16.4 ± 7.68	21.3 ± 13.7	0.004	16.6 ± 8.08	18.5 ± 9.95	0.268
Cost (CNY)	41,273.2 ± 9915.1	48,869.8 ± 29,867.7	0.041	41,273.2 ± 9385	49,443.6 ± 34,212	0.097
Death(%)	1(1.5%)	4(1.7%)	1.000	1(1.9%)	1(1.9%)	0.315
DGE(%)	13(19.4%)	41(17.0%)	0.649	10(19.2%)	2(3.8%)	0.014
Severe DGE	12(17.9%)	21(8.7%)	0.031	9(17.3%)	0(0%)	0.003

### Risk factors for SDGE

In the univariate logistic regression analysis, surgical method, biliary leakage pancreatic fistula, and abdominal infection were associated with postoperative SDGE. By contrast, age, gender, diabetes, albumin, anastomosis, bleeding, and operation time did not have significant effects. The significant risk factors determined in the univariate analysis and diabetes (*p* = 0.059) were used in a multivariate logistic regression analysis. We found that diabetes, surgical method, biliary leakage, and abdominal infection were significant independent risk factors for SDGE (Table 4).

**Table 4:** Risk factors for SDGE according to Logistic regression model

Factors	Subgroup	SDGE (*n* = 247)					
		Univariate analysis			Multivariate analysis		
		HR	95%CI	*p*	HR	95%CI	*p*
Age	< 60	1		0.901			
	≥ 60	0.95	0.43–2.12				
Gender	Female	1		0.320			
	Male	1.55	0.65–3.71				
Diabetes	No	1		0.059	1		0.045
	Yes	2.50	0.97–6.47		3.00	1.03–8.78	
Albumin	< 30	1		0.702			
	≥ 30	1.50	0.19–12.01				
Surgery	PrPD	1		0.010	1		0.002
	PPPD	3.00	1.30–6.95		4.78	1.78–12.86	
Anastomosis	PO	1		0.150			
	PG	1.81	0.81–4.04				
Biliary leakage	No	1		0.001	1		0.002
	Yes	6.07	2.15–17.15		7.01	2.07–23.78	
Pancreatic fistula	No	1		0.030	1		0.542
	Yes	2.57	1.10–6.02		0.71	0.24–2.11	
Bleeding	No	1		0.359			
	Yes	2.12	0.43–10.54				
Abdominal infection	No	1		0.000	1		0.004
	Yes	4.82	2.10–11.04		4.32	1.61–11.59	
Operation time	< 6 h	1		0.643			
	≥ 6 h	1.63	0.21–13.00				

### Development of clinical nomogram for SDGE

**Fig. 1:**
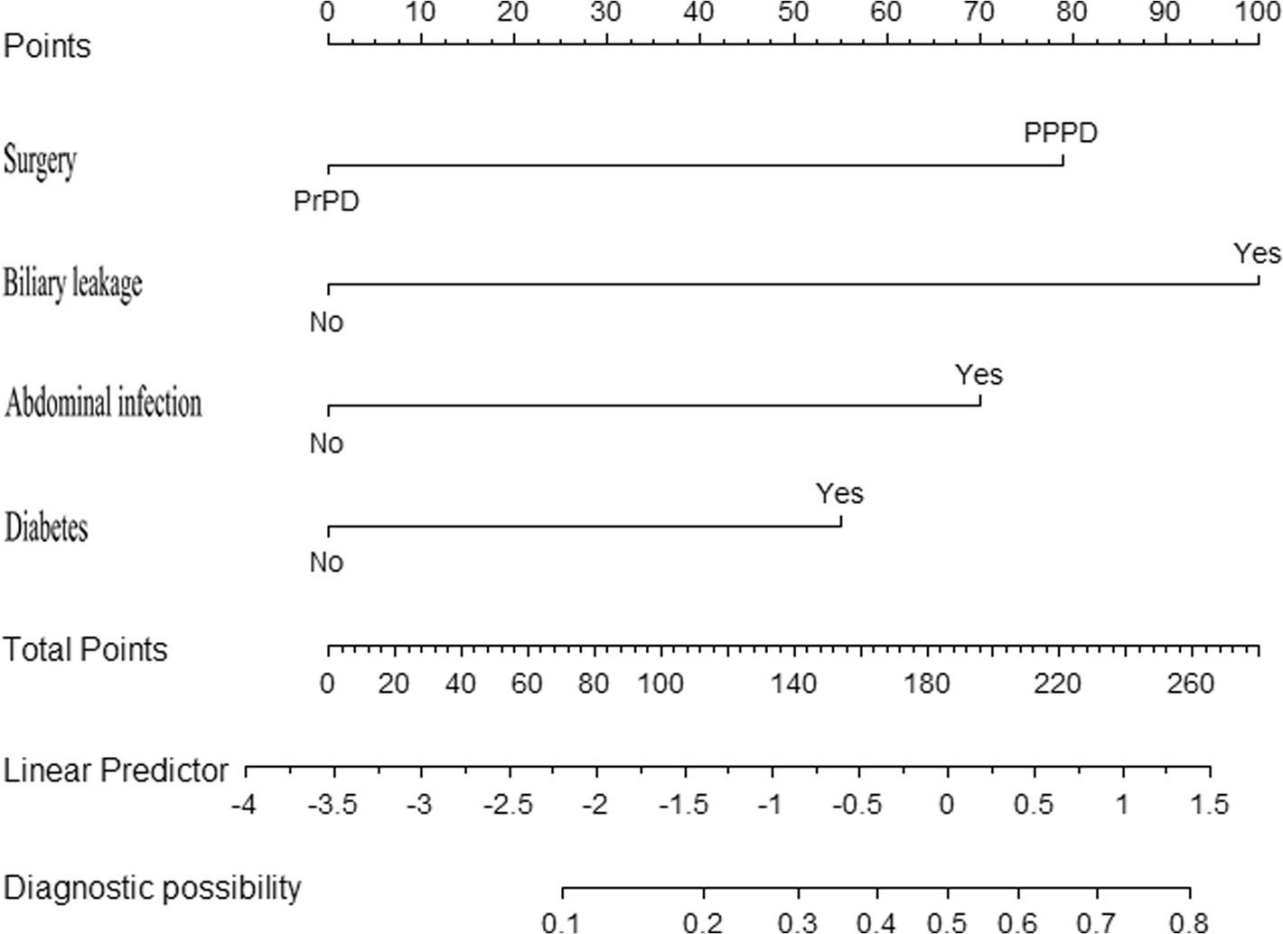
Nomogram predicting the probability of SDGE. Each risk factor corresponded to a point by drawing a line straight upward to the points axis (79 points for PPDD, 100 points for biliary leakage, 70 points for abdominal infection, and 55 points for diabetes). The sum of the points located on the total points axis represented the probability of SDGE by drawing a line straight down to the incidence axis

**Fig. 2:**
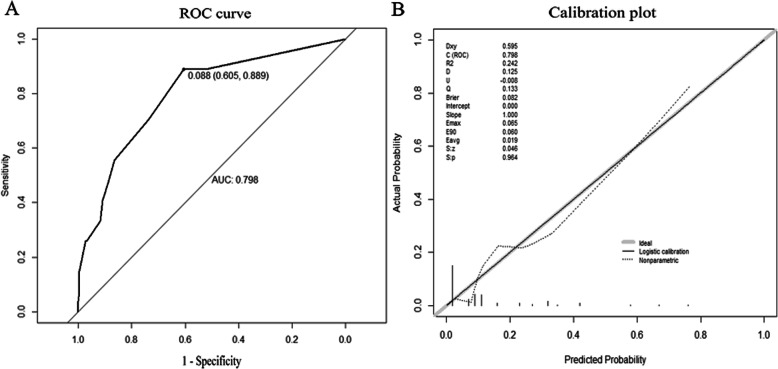
discrimination and calibration of the predictive model according to the development set: **a**. ROC curves; **b**. calibration plot

All patients were randomly divided into two groups: a development set (80%) and a validation set (20%). Detailed baseline characteristics of the development and validation sets are displayed in Table 5. Based on the results of the multivariate logistic regression analysis, diabetes, surgical method, biliary leakage, and abdominal infection were used to develop a prediction model and to generate a nomogram predicting the probability of SDGE (Fig. 1). The predictive accuracies of the nomogram calculated using AUC were 0.798 (95% CI: 0.708–0.887) (Fig. 2a) for the development set and 0.726 (95% CI: 0.486–0.966) for the validation set (Supplementary Fig. 1A). The calibration plot using the Hosmer–Lemeshow test revealed good agreement between predicted probability and observed outcome for the development set (*p* = 0.98, Fig. 2b) and the validation set (*p* = 0.99, Supplementary Fig. 1B).

**Table 5:** Baseline characteristics of the development and validation set

Factors	Subgroup	development set (*n =* 247) No of patient (%)	Validation set(*n* = 61) No of patient (%)
Age	≥ 60	140 (56.7)	18 (29.5)
	< 60	107 (43.3)	43 (70.5)
Gender	Male	152 (61.5)	47 (77.0)
	Female	95 (38.5)	14 (23.0)
Diabetes	Yes	34 (13.8)	7 (11.5)
	No	213 (86.2)	54 (88.5)
Albumin	≥ 30	234 (94.7)	60 (98.4)
	< 30	13 (5.3)	1 (1.6)
Surgery	PPPD	52 (21.1)	15 (24.6)
	PrPD	195 (78.9)	46 (75.4)
Anastomosis	PG	142 (57.5)	35 (57.4)
	PO	105 (42.5)	26 (42.6)
Biliary leakage	Yes	19 (7.7)	1 (1.6)
	No	228 (92.3)	60 (98.4)
Pancreatic fistula	Yes	51 (20.6)	11 (18.0)
	No	196 (79.4)	50 (82.0)
Bleeding	Yes	10 (4.0)	0 (0)
	No	237 (96.0)	61 (100)
Abdominal infection	Yes	67 (27.1)	10 (16.4)
	No	180 (72.9)	51 (83.6)
PBD	No	11 (4.5)	6 (9.8)
	Yes	236 (95.5)	55 (90.2)
Operation time	≥ 6 h	233 (94.3)	60 (98.4)
	< 6 h	14 (5.7)	1 (1.6)
SDGE	Yes	27 (10.9)	6 (9.8)
	No	220 (89.1)	55 (90.2)

*PBD* preoperative biliary drainage, *PG* pancreaticogastrostomy, *PO* Pancreatojejunostomy

## Discussion

The major findings of this study were that PPPD was significantly associated with an increased rate of postoperative DGE, especially severe DGE. There was no significant difference in length of hospital stay and hospitalization costs between PPPD and PrPD groups after adjustment for possible confounders. In addition to surgical method, biliary leakage, abdominal infection, and diabetes were also independent risk factors for SDGE. Moreover, We generated a nomogram and validated it to predict the probability of SDGE. For example, a patient with diabetes (55 points) who underwent PPPD (79 points) had postoperative abdominal infection (70 points) but no biliary leakage (0 points). This patient had a total score of 204 points. The predicted probability of SDGE would be approximately 55%.

DGE is one of the most common postoperative complications of pancreaticoduodenectomy. The reported incidence of DGE varied widely and remained controversial in various medical centers according to the definitions from ISGPS. In some studies [7, 23, 24], nearly one-third of patients suffered DGE after PD. In our study, the overall incidence of DGE was 17.5%. Although DGE is not a life threatening complication, it causes severe discomfort and decreases the quality of life postoperatively; it is also considered to increase the length of hospital stay and hospital costs [1].

Several retrospective studies and some high-quality randomized controlled trials (RCTs) have been reported. An RCT by Kawai et al. [1] found that the incidence of DGE was significantly higher in the PPPD group than in the PrPD group (17.2% versus 4.5%). Another RCT by Matsumoto et al. [2] showed that although the incidence of DGE in the PPPD group was higher than that of the PrPD group(20% versus 12%), the difference was not statistically significant. Two RCTs conducted by Seiler et al. [25] and Tran et al. [26] found that the classic Whipple procedure had no effect on reducing the incidence of DGE when compared with PPPD. However, the definition of DGE was not assessed according to the ISGPS. A retrospective study by Fujii et al. [27] showed that the incidence of DGE was significantly higher in the PPPD group (27.3%) than in the PrPD group (5.4%) and Nanashima et al. [28] found patients in PPPD group were more likely to have severe DGE. There have also been meta-analyses published recently. The meta-analysis by Wu et al. [11] included 27 studies involving 2599 patients; they found that PrPD reduced DGE incidence but increased blood loss when compared with PPPD, and the lengths of hospital stay were similar in the two groups. Another meta-analysis by Yang et al. [12] included eight RCTs with a total of 622 patients; they also presented similar results that the PPPD group had a higher rate of DGE (RR = 2.35, 95% CI: 1.06–5.21). However, the most recent meta-analysis by Hanna et al. [8] found that there was no significant difference between PPPD and classic PD. Therefore, disputes still exist.

The pathogenesis and mechanism of DGE after PD remains under investigation. Several factors are thought to be related to the occurrence of DGE: possible ischemia of pylorus and duodenum after surgery [29, 30]; gastricatony caused by denervation of pylorus ring or pylorospasm [31, 32]; gastric dysrhythmias caused by other complications [33, 34]; and the lack of gastrointestinal hormone [33]. More basic science studies are needed to determine the underlying mechanisms.

The anatomical configurations of reconstruction are thought to be important for gastric emptying. Kurahara et al. [35] found that the overall incidence of DGE in the antecolic group was significantly lower than that of the retrocolic group. However, another two RCTs found no difference between antecolic reconstruction and retrocolic reconstruction [36, 37]. Barakat et al. found that proximal Roux-en-y gastrojejunal anastomosis reduced the occurrence of DGE [38]. In the present study, all patients underwent antecolic reconstruction. Although the proportion of binding pancreaticogastrostomy was different between PPPD and PrPD groups, propensity score matching helped modify the possible confounding factors. Hanna et al. [8] found there was no difference between pancreaticogastrostomy and gastrojejunostomy.

In the present study, we found that the PPPD group had a significantly higher rate of postoperative pancreatic fistula and less blood loss. There were no differences in the occurrence rate of intra-abdominal infection, biliary leakage, postoperative bleeding, or mortality between the PPPD and PrPD groups. Though we did not analyze long-term survival or postoperative nutritional status, these studies revealed no differences in long-term survival or nutritional status between the PPPD and PrPD groups [1, 2, 12, 39, 40].

Although the occurrence of DGE was higher in the PPPD group than in the PrPD group, there were no differences in the length of hospital stay or hospital costs between the two groups in our study. We think the reasons for this are as follows. First, the patients were identified from 2009 to 2018, but we did not follow the contemporary ERAS type management of patients strictly from 2015. We usually engaged in a long observation to ensure the safety of patients in the hospital. Second, DGE is usually cured using conservative treatments, and the costs of hospitalization and nursing were low according to the special health care system in China. Therefore, the average hospital stay was quite long. In our opinion, it is reasonable to believe that DGE could be unpleasant for patients and could increase the length of hospital stay when the ERAS protocol is performed strictly.

A randomized trial could provide a more reliable conclusion. The randomized method was used to prevent selective bias; the comparability of the two groups was better; and statistical results were more convincing. In our study, although the choice to perform PPPD or PrPD was determined by the surgeon at random rather than being related to the extent of tumor or some other factor, we did not use a strict randomization approach; therefore, there may have been subjective selection bias on the part of the surgeon. For this reason, propensity score matching was applied to build a randomized experiment-like situation and to decrease the influence of selection bias.

Our study has several advantages. First, to our knowledge, this is the first nomogram to predict the probability of SDGE after pancreaticoduodenectomy. SDGE usually requires an adjustment of clinical management. We also used internal validation to verify the good specificity and calibration of model; Second, propensity score matching was used to control for confounding biases to construct a randomized experiment-like situation; Third, this study had a large sample size, and we believe our experience adds meaningful data to the existing literature.

We acknowledge that this study also has several limitations. First, this study design was observational. Although many measured confounders were adjusted for by propensity score matching, the operation assignment was not randomized, and the results may be biased by other unmeasured factors; Second, we did not strictly follow the contemporary ERAS type management of patients. If we did not routinely place naso-gastric tubes or maintain the patients without enteral intake for 3 days, the differences between surgical methods may have changed. Further studies are needed to clarify this point; Third, our predictive model included post-operative complications as predictors of SDGE. When patients had these complications, their management would be more important than SDGE. This situation decreased the utility of the instruments. We will attempt to identify other pre-operative factors to predict SDGE in the future. Finally, we did not measure long-term survival because the patients with malignant tumors and benign lesions were combined.

## Conclusions

PPPD increases the incidence of DGE. Biliary leakage, abdominal infection, and presence of diabetes were independent risk factors for SDGE. We created a simple nomogram for clinicians to make a preliminary estimation of the probability of SDGE. Further prospective studies are recommended to validate the model externally.

## Supplementary information

**Additional file 1.** Supplementary Table 1. The association between anastomotic pattern of the pancreas and other clinical characteristics

**Additional file 2:.** Supplementary Fig. 1 discrimination and calibration of the predictive model according to the validation set: A. ROC curves; B. Calibration plot

## Data Availability

The datasets used and/or analysed during the current study are available from the corresponding author on reasonable request.

## Abbreviations

PPPD: Pylorus-preserving pancreaticoduodenectomy
SSPPPDL: Subtotal stomach-preserving pancreaticoduodenectomy
PrPD: Pylorus-resecting pancreaticoduodenectomy
DGE: Delayed gastric emptying
SDGE: Severe delayed gastric emptying
ISGPS: The International Study Group of Pancreatic Surgery
OR: Odds ratio
CI: Confidence interval
RCT: Randomized controlled trials
WBC: White blood cell
TB: Total bilirubin
PBD: Preoperative biliary drainage
PG: Pancreaticogastrostomy
PO: Pancreatojejunostomy
PS: Propensity scores
PTCD: Percutaneous transhepatic cholangial drainage
ENBD: Endoscopic nasobiliary drainage
