# Supplementary material for: Delayed gastric emptying after Pancreaticoduodenectomy: a propensity score-matched analysis and clinical Nomogram study
Source: BMC Surg. 2020 Jul 9;20:149. doi: 10.1186/s12893-020-00809-5 (PMC7346444; doi:10.1186/s12893-020-00809-5)
Supplement: Supplementary file 1 — Additional file 1. Supplementary Table 1. The association between anastomotic pattern of the pancreas and other clinical characteristics [file 12893_2020_809_MOESM1_ESM.docx]

Supplementary Table 1. The association between anastomotic pattern of the pancreas and other clinical characteristics

| Factors | pancreaticogastrostomy | pancreatojejunostomy | *p* |
| --- | --- | --- | --- |
| Gender |  |  | **0.003** |
| male | 102 | 97 |  |
| female | 75 | 34 |  |
| Diabetes mellitus |  |  | 0.132 |
| yes | 28 | 13 |  |
| No | 149 | 118 |  |
| Jaundice reduced |  |  | 0.535 |
| yes | 11 | 166 |  |
| No | 6 | 125 |  |
| Enteral nutrition |  |  | 0.934 |
| yes | 29 | 148 |  |
| No | 21 | 110 |  |
| Pancreatic fistula |  |  | 0.450 |
| yes | 33 | 144 |  |
| No | 29 | 102 |  |
| Biliary leakage |  |  | **0.001** |
| yes | 4 | 173 |  |
| No | 16 | 115 |  |
| Abdominal infection |  |  | 0.054 |
| yes | 37 | 140 |  |
| No | 40 | 91 |  |
| Bleeding |  |  | 0.627 |
| yes | 5 | 172 |  |
| No | 5 | 126 |  |
| Severe DGE |  |  | 0.064 |
| yes | 14 | 19 |  |
| No | 163 | 112 |  |
| Death |  |  | 0.167 |
| yes | 1 | 176 |  |
| No | 4 | 127 |  |
