# Supplementary figures and images for: Delayed gastric emptying after Pancreaticoduodenectomy: a propensity score-matched analysis and clinical Nomogram study
Source: BMC Surg. 2020 Jul 9;20:149. doi: 10.1186/s12893-020-00809-5 (PMC7346444; doi:10.1186/s12893-020-00809-5)

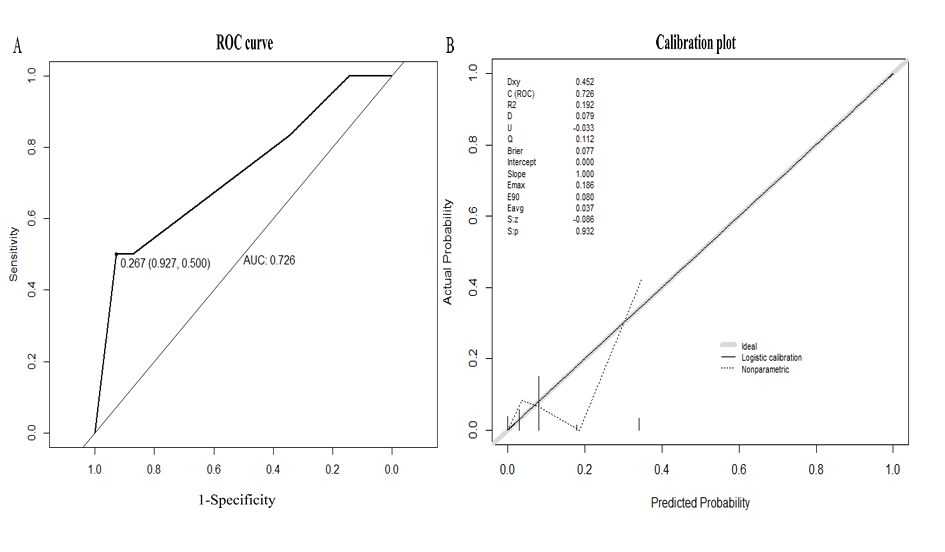

Supplement: Supplementary file 2 — Additional file 2:. Supplementary Fig. 1 discrimination and calibration of the predictive model according to the validation set: A. ROC curves; B. Calibration plot [file 12893_2020_809_MOESM2_ESM.tif]
